# Role and prognostic value of oncostatin M and its receptor OSMR in acute myeloid leukemia, myeloproliferative neoplasms and non-hematological malignancies

**DOI:** 10.3389/fonc.2025.1636570

**Published:** 2025-09-17

**Authors:** Jean-Pierre Lévesque, Kavita Bisht, Kylie A. Alexander, Ingrid G. Winkler

**Affiliations:** Mater Research – The University of Queensland, Woolloongabba, QLD, Australia

**Keywords:** oncostatin M, prognosis factor, tumor microenvironment, acute myeloid leukemia, myeloproliferative neoplasm, glioblastoma, glioma, pancreatic adenocarcinoma

## Abstract

The oncostatin M receptor (OSMR) has recently emerged as an adverse prognostic factor in acute myeloid leukemia (AML) and several non-hematological malignancies. In this perspective, we discuss how oncostatin M (OSM) and its receptor OSMR regulate tumor cells as well as mesenchymal and endothelial cells, which are key components of hematopoietic stem cell and tumor stem cell niches, and how these mechanisms could explain the poor prognosis associated with high expression of OSM and OSMR in hematological and non-hematological malignancies.

## 1 Introduction

High expression of oncostatin M (OSM) receptor OSMR has recently emerged as a poor prognosis factor in several malignancies such as acute myeloblastic leukemia (AML), gliomas, pancreatic, gastric and kidney carcinomas. In this perspective, we perform a larger in silico survey of the prognostic value of high *OSMR* and *OSM* transcripts in 33 malignancies and discuss the mechanisms involved in the adverse effects of high OSM-mediated signaling in malignant cells and the tumor microenvironment, and potential treatments to target this signaling pathway.

OSM is an inflammatory cytokine of the interleukin-6 (IL-6) family. OSM binds to two different receptors made up of glycoprotein-130 (GP130, gene *IL6ST* in humans *Il6st* in mice) complexed with either transmembrane OSM receptor (OSMR, gene *OSMR* in humans or *Osmr* in mice) or with leukemia inhibitory factor receptor (LIFR) (1, 2). Both OSMR and LIFR have very long intracellular domains enabling the docking of many kinases and adaptors, eliciting similar but not identical signaling in response to OSM (3, 4). OSM is mostly produced by activated myeloid cells (granulocytes, monocytes and macrophages) as well as all cells of the osteoblast lineage (3, 5, 6), and astrocytes, neurons, microglia in the brain (7). In contrast, its receptor OSMR is not expressed by leukocytes or hematopoietic stem and progenitor cells (HSPC) but expressed by cells of the mesenchymal and endothelial lineages in the bone marrow (6) and other tissues. OSM is a regulator of both hematopoiesis and skeleton homeostasis mostly via the OSMR: GP130 receptor complex (6) and plays important roles in inflammatory responses in skeletal (8, 9) and cardiac (10) muscles, lung (11), liver (12, 13), intestine (14), adipose (15), skin (16, 17) and joints (18). Adding to the complexity of OSMR protein roles, OSMR protein complexed with the IL-31 receptor α chain IL31RA acts as a receptor for IL-31 (19). Recent literature now highlights important roles of both OSMR and OSM in the pathogenesis and response to treatment of several malignancies as detailed below.

## 2 OSM and OSMR in hematological malignancies

In a recent paper, high throughput proteomic analysis on more than 550 newly diagnosed AML patients demonstrated that high plasma concentration of soluble OSMR protein was a strong independent predictor of poor survival and early mortality (20). These clinical data suggest an oncogenic role of OSMR-mediated signaling and are consistent with the anti-proliferative effect of *OSMR* gene inactivation in mice and humans. Indeed, *Osmr* ^-/-^ mice display mild anemia and thrombocytopenia (21–23) with decreased HSPC cycling in the bone marrow, increased HSPC chemotactic response, increased HSPC mobilization into the blood in response to G-CSF or CXCR4 antagonists, as well as decreased expression of genes associated with cell cycling, lipid metabolism, and erythropoiesis in hematopoietic stem cells (HSC) (5). Another recent report has shown that biallelic loss-of-function of the *OSM* gene in humans causes profound anemia, thrombocytopenia and neutropenia (24). Therefore, OSM and OSMR-mediated signaling contributes to increased HSPC cycling and retention within the bone marrow, enabling sufficient erythropoiesis and thrombopoiesis output. Interestingly, the hematopoietic effects of OSMR are mediated via the hematopoietic environment as *OSMR* mRNA is undetectable to very low in mouse and human HSPC (5) or in AML blasts (20). Reville et al. highlighted that high OSMR protein production and transcript expression was limited to mesenchymal stromal cells (MSCs) while undetectable in AML blasts in cultures of leukemic marrow aspirates (20). However, their culture system did not enable the survival and growth of bone marrow endothelial cells, which similar to MSCs, express high levels of OSMR transcripts and protein (5, 6) and are key functional regulatory elements of HSC niches and leukemia stem cell niches (25–30). Therefore, the source of sOSMR protein in the blood of AML patients could be endothelial cells as well as MSCs and the deregulation of these niche cell types in the bone marrow may be involved in the mechanisms of poorer outcome in patients with high sOSMR protein.

**Figure 1:**
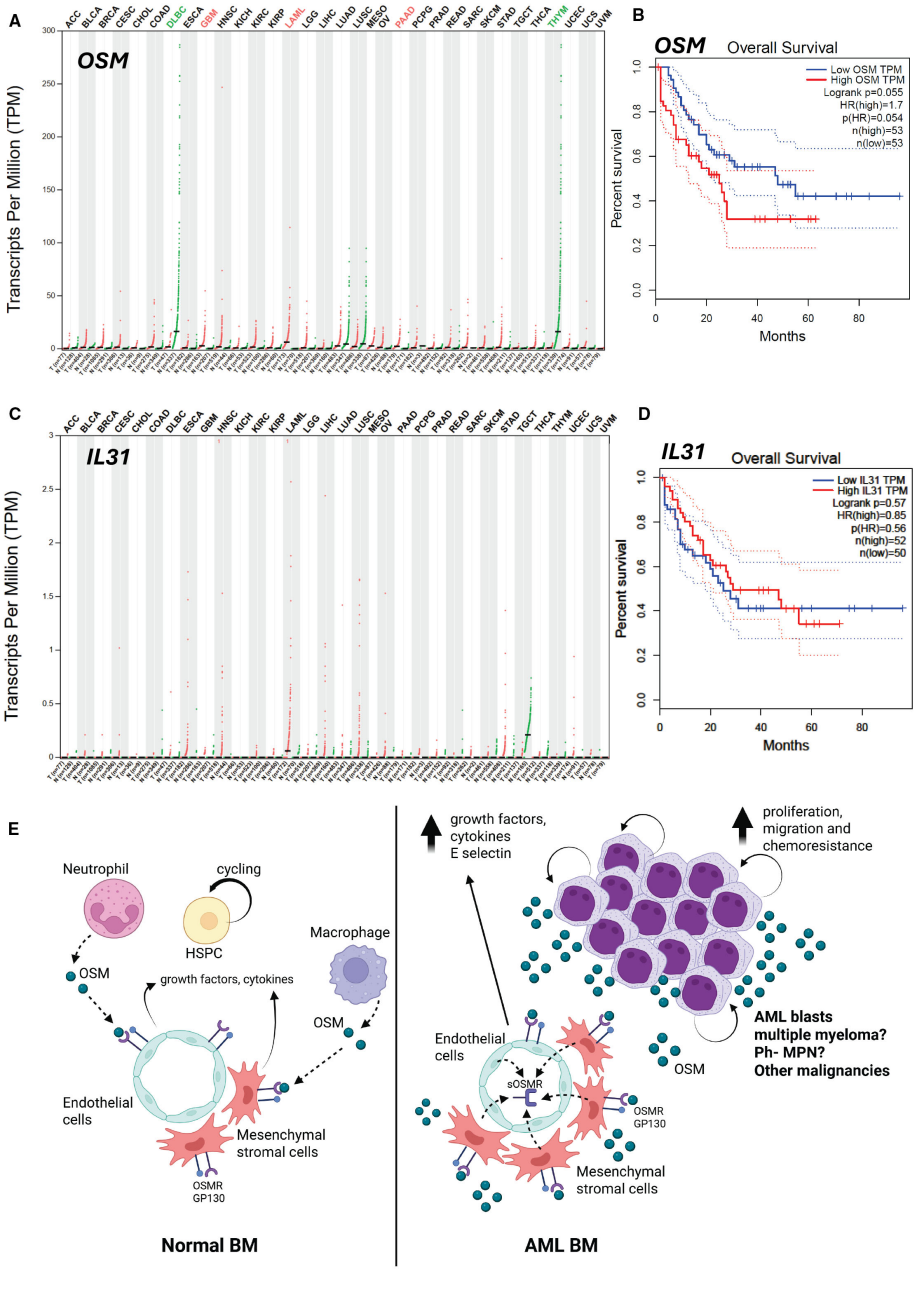
Expression of transcripts for OSMR ligands OSM and IL-31 with overall survival plots of AML patients with highest and lowest expression *OSM* and *IL31* transcripts. **(A)** *OSM* transcript quantification in transcripts per million in various tumors (red dots) versus paired normal tissues (green dots) extracted from the TCGA database stratified as adrenocortical carcinoma (ACC), bladder urothelial carcinoma (BLCA), breast invasive carcinoma (BRCA), cervical squamous cell carcinoma and endocervical adenocarcinoma (CESC), cholangial carcinoma (CHOL), colon adenocarcinoma (COAD), diffuse large B-cell lymphoma (DLBC), esophageal carcinoma (ESCA), glioblastoma multiform (GBM), head and neck squamous cell carcinoma (HNSC), kidney chromophobe carcinoma (KICH), kidney renal clear cell carcinoma (KIRC), kidney renal papillary cell carcinoma (KIRP), acute myeloid leukemia (LAML), lower grade glioma (LGG), hepatocellular carcinoma (LIHC), lung adenocarcinoma (LUAD), lung squamous cell carcinoma (LUSC), mesothelioma (MESO), ovarian serous cystadenocarcinoma (OV), pancreatic adenocarcinoma (PAAD), pheochromocytoma and paraganglioma (PCPG), prostate adenocarcinoma (PARD), rectum adenocarcinoma (READ), sarcoma (SARC), skin cutaneous melanoma (SKCM), stomach adenocarcinoma (STAD), testicular germ cell tumor (TGCT), thyroid carcinoma (THCA), thymoma (THYM), uterine corpus endometrial carcinoma (UCEC), uterine carcinosarcoma (UCS) and uveal melanoma (UVM). Bars show median value for each tumor (T) and normal tissue (N) with number of samples indicated. Significant differences in *OSM* transcripts per million between malignant and paired normal tissues are indicated with colored malignancy abbreviation above the chart in red for *OSM* overexpressed in tumor versus paired normal tissue and in green for *OSM* under-expressed in malignant versus paired normal tissue. **(B)** Kaplan-Meier plots of overall survival of AML patients with 50% highest (red plot) and 50% lowest *OSM* (blue plot) transcripts, log-rank test p value, hazard ratio (HR), significance of hazard ratio p(HR) and number of patients are indicated on the plot (n=106). **(C)** *IL31* transcript in various tumors (red dots) versus paired normal matching tissues (green dots) extracted from the TCGA database and stratified as described in **(A)**. **(D)** Kaplan-Meier plots of overall survival of AML with 50% highest and 50% lowest *IL31* transcripts. **(E)** Model of OSM and OSMR effects on normal hematopoiesis (left side) and AML (right side). In normal bone marrow, low level of OSM is released by myeloid cells stimulating basal production of cytokines/growth factors by MSCs and endothelial cells expressing OSMR/GP130 receptor complex and forming HSPC niches. In the leukemic bone marrow, malignant AML blasts express and release high levels of OSM, stimulating MSCs and endothelial cells via the OSMR/GP130 receptor complex they express, to release excessive amounts of growth factors, cytokines, and sOSMR protein as well as boost endothelial E-selectin expression, all contributing to enhanced AML blast proliferation, migration and chemoresistance. Similar effects may occur in multiple myeloma, Philadelphia-negative MPN, and solid tumors. Panels **(A–D)** were generated by using the GEPIA website in April 2025 (32).

As the OSMR ligand OSM is expressed by myeloid cells (particularly neutrophils and macrophages) and osteoblasts in the bone marrow (3, 5), we plotted the expression of *OSM* transcripts in the 9,736 tumor and 8,587 normal tissue samples contained in The Cancer Genome Atlas (TCGA) database using Gene Expression Profiling and Interactive Analysis (GEPIA) website (31, 32) (**Figure 1A**). *OSM* transcripts were significantly higher in AML samples than normal bone marrow samples (“LAML” in **Figure 1A**). Overall survival (OS) Kaplan-Meier plot from AML patients with 50% highest content and 50% lowest content in *OSM* transcripts is shown in **Figure 1B**. Although the p values of p=0.055 for differences were just above the significance threshold of p=0.05, AML patients with highest levels of *OSM* transcript had a trend to shorter OS with a 1.7 hazard ratio compared to AML patients with lowest *OSM* transcripts. Considering that high sOSMR protein is significantly associated with poorer prognosis in AML (20), confirmation of a significant association between poor prognosis and high *OSM* transcripts in a larger cohort of AML patients is warranted. In regard to the alternative OSMR ligand interleukin-31 (IL-31), *IL31* transcripts were detected at least 2 orders of magnitude lower compared to *OSM* transcripts (**Figure 1C**) and there was no difference in OS between high and low *IL31* expressing AML with the 50% cut-off (**Figure 1D**). Therefore, it is likely that the adverse effect of high *OSM* transcript and sOSMR protein in AML is mediated via its ligand OSM acting indirectly via bone marrow mesenchymal and endothelial cells which express both OSMR with its co-receptor GP130 (5, 22, 23) rather than IL-31-mediated signaling. One of the adverse effects of OSM-OSMR interaction may be mediated through its induction of E-selectin expression by endothelial cells (33) as E-selectin mediated signaling in AML stem cells increases their resistance to the cytotoxic effects of chemotherapy (30) (see model in **Figure 1E**).

In respect to other hematological malignancies, *OSM* mRNA are reportedly high in Philadelphia-negative myeloproliferative neoplasm (Ph^-^ MPN) cells with a constitutively active JAK2 tyrosine kinase mutations such as *JAK2* ^V617F^ (34), with increased OSM protein concentration in Ph^-^ MPN patient bone marrow cells and blood plasma (34). Furthermore cytokine production by primary fibroblasts from MPN patients is increased in response to OSM via OSMR expressed by fibroblasts (34). Therefore, sOSMR and its ligand OSM may also have prognostic value in Ph^-^ MPN. This hypothesis is consistent with the following findings: 1) Transduction of a *JAK2* ^V617F^ mutant in myeloid cell lines dramatically increases OSM expression (34). 2) OSM promotes HSPC proliferation, erythropoiesis and thrombopoiesis *in vivo* (5, 21) indirectly via bone marrow niche cells that express OSMR (5, 6). 3) In a mouse model of lymphoproliferative neoplasm driven by activating mutation of the FLT3 tyrosine kinase domain (FLT3-TKD), co-transduction with OSM cDNA switched the lymphoproliferative FLT3-TKD neoplasm to a myeloproliferative neoplasm suggesting that OSM is a potent inducer of a pro-myeloid niche (35). 4) Transplantation of mouse HSCs overexpressing OSM resulted in fatal myelofibrosis (36), a common adverse evolution of Ph^-^ MPNs. Further along this latter point, it has recently emerged that activating JAK2^V617F^ mutation upregulates the expression of the “don’t eat me” antigen CD24 at the surface of neutrophils enabling the accumulation of old senescent neutrophils to escape efferocytosis and accumulate in the bone marrow promoting evolution to myelofibrosis (37). As neutrophils are a major source of OSM protein in the bone marrow (5, 6) and OSM overexpression triggers myelofibrosis (36), accumulation of OSM secreted by senescent JAK2^V617F^ neutrophils may be a driver of the evolution of polycythemia vera (the most frequent clinical manifestation of MPNs with JAK2^V617F^ driving mutation) to myelofibrosis which has worse clinical outcome.

In respect to lymphoid neoplasms, high sOSMR (19) and OSM (38) protein concentrations have also been reported in the serum of multiple myeloma patients. However, high plasma OSM had no significant prognostic value in newly diagnosed multiple myeloma patients (38) and the potential prognosis value of sOSMR concentrations in multiple myeloma has not been reported.

## 3 OSM and OSMR in non-hematological malignancies

**Figure 2:**
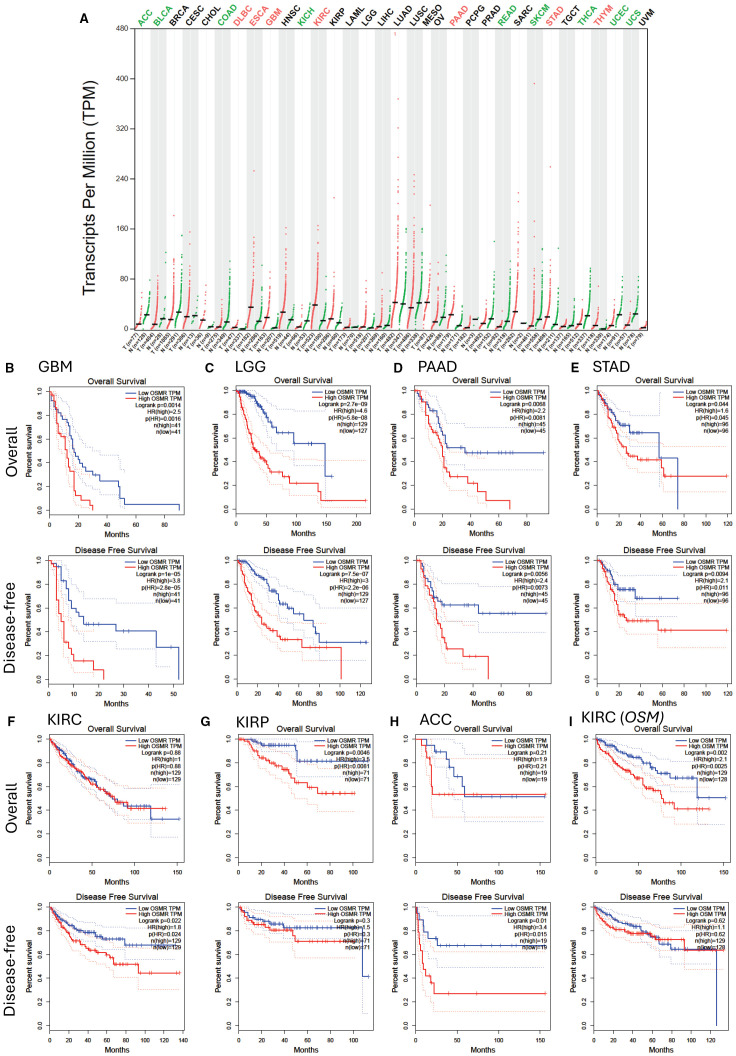
*OSMR* transcript expression and patient survival of highest and lowest 25% *OSMR* transcript expressing tumors. **(A)** *OSMR* transcript quantification in transcripts per million in various tumors (red dots) versus paired normal tissues (green dots) extracted from the TCGA database stratified as described in **Figure 1A**. Significant differences in *OSMR* transcript per million between malignant and paired normal tissues are indicated with colored malignancy abbreviations above the chart with red for *OSMR* transcripts significantly overexpressed in tumor versus paired normal tissue and green for *OSMR* transcripts significantly under-expressed in malignant versus paired normal tissue. **(B–H)** Kaplan-Meier plots of overall and disease-free survival of patients with 25% highest *OSMR* transcript (red curves) versus lowest 25% *OSMR* transcript (blue curves) for **(B)** glioblastoma multiform (GBM, n=162), **(C)** low grade glioma (LGG, n=514), **(D)** pancreatic adenocarcinoma (PAAD, n=178), **(E)** stomach adenocarcinoma (STAD, n=384), **(F)** kidney renal clear cell carcinoma (KIRC, n=516), **(G)** kidney papillary cell carcinoma (KIRP, n=280), and **(H)** adrenocortical carcinoma (ACC, n=76). **(I)** Kaplan-Meier plots of overall and disease-free survival of patients with 25% highest *OSM* transcript (red curves) versus lowest 25% OSM transcript (blue curves) for kidney renal clear cell carcinoma (KIRC). Log-rank test p value, hazard ratio (HR), significance of hazard ratio p(HR) and number of patients are indicated on each plot. Panels were generated by using the GEPIA website in April 2025 (32).

Endothelial cells (39, 40) and mesenchymal cells (41) are important players in cancer stem cell niches within solid tumors. As OSMR is expressed by these cells (5, 22, 23) as well as some mucosal and glandular epithelial cells (42), OSMR and OSM may also play important roles in several non-hematological malignancies by directly acting on tumor cells or indirectly via endothelial and mesenchymal cells such as fibroblasts in the tumor environment. In support of this, high *OSMR* transcript content is a poor prognosis factor in glioblastoma (43, 44), pancreatic adenocarcinoma (45), and gastric cancer (46). Analysis of *OSMR* transcript expression in all tumor types and corresponding healthy tissues in the TCGA database (**Figure 2A**) showed *OSMR* transcripts are significantly more expressed in tumors than the paired normal tissues in esophageal carcinoma (ESCA), glioblastoma multiform (GBM), kidney renal clear cell carcinoma (KIRC), pancreatic adenocarcinoma (PAAD), stomach adenocarcinoma (STAD) and thymoma (THYM). Lower grade glioma (LGG) had higher expression of *OSMR* when compared to healthy tissue, but this was not significant. As samples were generally higher in solid tumor datasets compared to AML, we used a more stringent quartile cut-off and plotted OS and disease-free survival (DFS) from the 25% highest *OSMR* transcript expressing tumors (High OSMR) and 25% lowest *OSMR* expressing tumors (Low OSMR). GBM, LGG, PAAD and STAD with highest *OSMR* transcripts had significantly worse OS, DFS and hazard ratios than the same tumors with lowest *OSMR* expression (**Figures 2B–E**). High *OSMR* transcripts in KIRC had no effect on OS but significantly poorer DFS (**Figure 2F**). Among tumor types with similar *OSMR* transcripts compared to paired normal tissue, kidney renal papillary cell carcinoma (KIRP) had significantly worse OS but no difference in DFS (**Figure 2G**). Unexpectedly, adrenocortical carcinoma (ACC), had significantly less *OSMR* transcripts compared to paired normal tissue (**Figure 2A**) but patients with high *OSMR* transcripts had significantly poorer DFS and equivalent OS (**Figure 2H**). We could not find any significant difference in OS and DFS between high and low *OSMR* expression in all other tumor types contained in the TCGA database (result not shown).

Reciprocally, *OSM* mRNA expression was significantly higher in GBM, PAAD and AML compared to corresponding healthy tissue (**Figure 1A**). OS was significantly worse in KIRC with high *OSM* transcripts but there was no significant difference in DFS (**Figure 2I**). There was no difference in OS or DFS between high and low *OSM* transcript levels in GBM or PAAD patients (result not shown). We also checked that these findings were similar with a median cut-off (50%) for *OSMR* and *OSM* transcripts (**Supplementary Figure 1**). This was true except for KIRP and ACC which did not show significant difference for OS or DFS with a 50% cut-off.

The poorer prognosis of tumors with high *OSMR* transcripts is consistent with previously reported effects of *OSM* or *OSMR* gene knock-down on these tumor types. For instance, OSM protein has been reported to increase migration, invasiveness and mesenchymal phenotype of GBM cell lines (43), and OSMR protein can translocate to the mitochondrial matrix to protect GBM stem cells from irradiation (44). Likewise OSM has been found to enhance tumor growth and epithelial-to-mesenchymal transition of pancreatic ductal adenocarcinoma cell lines *in vivo* in immunodeficient mice via OSMR (45). Additionally, OSM produced by macrophages has been found to induce an inflammatory phenotype in PAAD-associated fibroblasts providing a pro-tumorigenic environment in PAAD (47). OSM also promotes the proliferation of gastric cancer cells *in vitro* and *in vivo* in Nude mice (46). Finally, OSM has been reported to increase migration, invasiveness and epithelial-to-mesenchymal transition of clear cell renal carcinoma cell lines *in vitro* (48). Importantly, deletion of the *VHL* gene specifically in kidney tubule cells in mice (approximately 70% of clear cell renal carcinoma have inactivating mutation of *VHL*) induces production of OSM which in turn pushes kidney endothelial cells into an endothelial-to-mesenchymal transition to support *VHL*-defective tubule cell transformation and promote macrophage polarization towards a tissue-supportive M2-like phenotype (49). This vicious loop between OSM-expressing *VHL*-defective tubule cells and activated endothelium expressing OSMR is functionally important as injection of neutralizing OSM antibodies into immuno-deficient mice transplanted with human clear cell renal carcinoma cells inhibited primary tumor vascularization, growth and metastasis (49).

## 4 Discussion

Kaplan-Meier plots in **Figures 1**, **2** and previous reports discussed above clearly indicate that the interaction of OSM with the OSMR: GP130 receptor complex plays a role in the pathobiology and response to treatment of a number of malignancies such as AML, MPNs, glioma and glioblastoma, pancreatic, gastric and renal carcinomas where high expression of *OSMR* or *OSM* transcripts has poor prognosis value. The effects of the OSM: OSMR interaction are multipronged as it can alter either the function of malignant stem cell niches as observed in malignant hematological stem cells that do not express OSMR such as AML or MPNs, or directly act on malignant cells that express OSMR with further support from tumor-associated fibroblasts and endothelial cells which also express OSMR.

As OSM protein is highly expressed by activated neutrophils, monocytes and macrophages, blood concentration of OSM protein may not be a reliable prognosis marker of cancer treatment outcome because OSM concentration is also elevated during infections, sepsis (50, 51), acute and chronic inflammation (14, 16, 52) or in response to therapeutic treatments with myelopoietic cytokines such as granulocyte colony-stimulating factor (5). On the other hand, OSMR is not expressed by leukocytes but by non-hematopoietic malignant cells, as well as endothelial and mesenchymal cells forming the tumor environment. As soluble sOSMR protein is produced by alternative splicing of the *OSMR* transcript in exon 8 (19), it is likely that sOSMR protein may be similarly increased in the plasma of patients with tumors expressing high levels of *OSMR* transcripts, although actual concentrations of plasma sOSMR were not measured in patient samples from the TCGA used for our in silico analysis.

## 5 Conclusion and perspectives

Blood sOSMR concentration may represent an easily measurable prognosis marker not only in AML as recently reported (20) but also in the other malignancies discussed in this perspective, which warrants further clinical studies for its prognostic potential. In regards of possible treatments of patients with high levels of sOSMR protein or *OSMR* or *OSM* gene expression, the main axis of OSMR-mediated signaling is via tyrosine phosphorylation and activation of transcription factors STAT1 and STAT3 (and in some cell types STAT5) via tyrosine kinases JAK1 and JAK2 (4, 6). Therefore, adjunct treatments with small non-selective JAK1 and JAK2 tyrosine kinase inhibitors such as ruxolitinib, tofacitinib and others (53) may improve outcome in these patients. Therapeutics that specifically target OSMR or OSM such as the humanized neutralizing anti-OSM monoclonal antibody GSK2330811 may also be of interest to treat AML patients with high sOSMR protein or *OSM* transcripts as GSK2330811 was found to induce thrombocytopenia, anemia and mild neutropenia in patients with diffuse cutaneous systemic sclerosis (54). Effectiveness of such neutralizing OSM antibodies in other malignancies in which high OSMR expression leads to poorer outcome remains to be established. In conclusion, elevated OSMR-mediated signaling is emerging as a poor prognosis factor in a number of malignancies such as AML, GBM, LGG, STAD, KIRC and KIRP. Consequently, *OSMR* gene expression and sOSMR protein blood concentration should be systematically measured in patients with these diseases and clinical trials should be undertaken to test efficacy of adjunct therapies with JAK1/2 inhibitors or neutralizing OSM antibodies to current best treatments.

## Data Availability

The original contributions presented in the study are included in the article/**Supplementary Material**. Further inquiries can be directed to the corresponding author.

## Glossary

ACC: adrenocortical carcinoma
AML: acute myeloblastic leukemia
BLCA: bladder urothelial carcinoma
CESC: cervical squamous cell carcinoma and endocervical adenocarcinoma
CHOL: cholangial carcinoma
COAD: colon adenocarcinoma
DFS: disease-free survival
DLBC: diffuse large B-cell lymphoma
ESCA: esophageal carcinoma
FLT-3: Fms-like tyrosine-protein kinase-3
GBM: glioblastoma multiform
GEPIA: Gene expression Profiling and Interactive Analysis
GP130: glycoprotein-130
HNSC: head and neck squamous cell carcinoma
HSC: hematopoietic stem cell
HSPC: hematopoietic stem and progenitor cell
IL: interleukin
JAK: Janus tyrosine-kinase
KICH: kidney chromophobe carcinoma
KIRC: kidney renal clear cell carcinoma
KIRP: kidney renal papillary cell carcinoma
LAML: acute myeloid leukemia
LGG: lower grade glioma
LIHC: hepatocellular carcinoma
LUAD: lung adenocarcinoma
LUSC: lung squamous cell carcinoma
MESO: mesothelioma
MPN: myeloproliferative neoplasm
MSC: mesenchymal stromal cell
OS: overall survival
OSM: oncostatin M
OSMR: oncostatin M receptor
sOSMR: soluble oncostatin M receptor protein
OV: ovarian serous cystadenocarcinoma
PAAD: pancreatic adenocarcinoma
PCGP: pheochromocytoma and paraganglioma
PARD: prostate adenocarcinoma
READ: rectum adenocarcinoma
SARC: sarcoma
SKCM: skin cutaneous melanoma
STAD: stomach adenocarcinoma
STAT: signal transducer and activator of transduction
TCGA: The Cancer Genome Atlas
TGCT: testicular germ cell tumor
THCA: thyroid carcinoma
THYM: thymoma
UCEC: uterine corpus endometrial carcinoma
UCS: uterine carcinosarcoma
UVM: uveal melanoma
VHL: von Hippel Lindau protein
