# Supplementary material for: Role and prognostic value of oncostatin M and its receptor OSMR in acute myeloid leukemia, myeloproliferative neoplasms and non-hematological malignancies
Source: Front Oncol. 2025 Sep 17;15:1636570. doi: 10.3389/fonc.2025.1636570 (PMC12484008; doi:10.3389/fonc.2025.1636570)
Supplement: Supplementary file 1 [file DataSheet1.pdf]

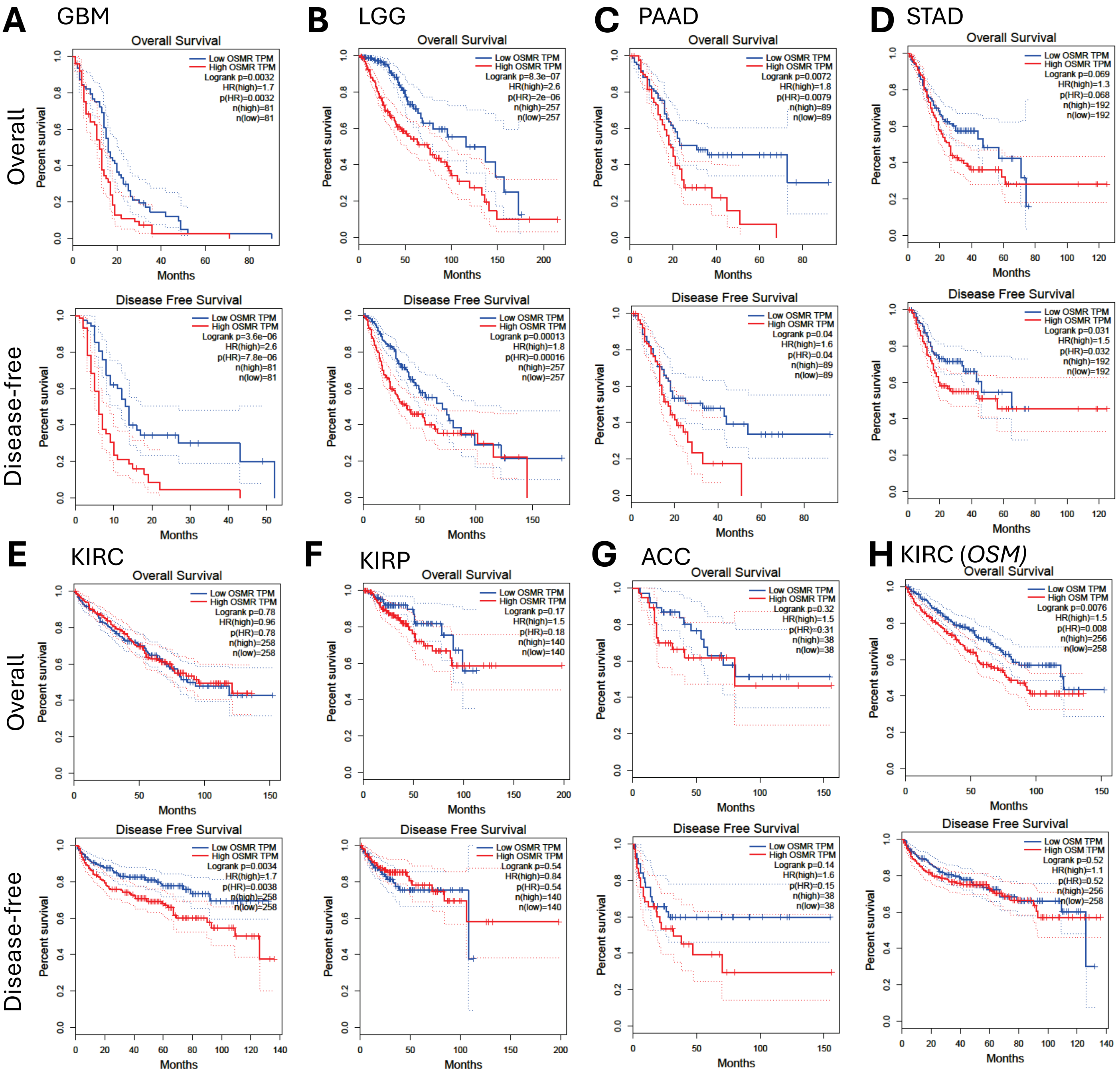

Supplementary Figure 1. *OSMR* transcript expression and patient survival of highest and lowest 50 % *OSMR* transcript expressing tumors**.** Kaplan-Meier plots of overall and disease-free survival of patients with 50% highest *OSMR* transcript (red curves) versus lowest 50% *OSMR* transcript (blue curves) for (B) glioblastoma multiform (GBM, n=162), (C) low grade glioma (LGG, n=514), (D) pancreatic adenocarcinoma (PAAD, n=178), (E) stomach adenocarcinoma (STAD, n=384), (F) kidney renal clear cell carcinoma (KIRC, n=516), (G) kidney papillary cell carcinoma (KIRP, n=280), and (H) adrenocortical carcinoma (ACC, n=76). **(I)** Kaplan-Meier plots of overall and disease-free survival of patients with 50% highest *OSM* transcript (red curves) versus lowest 50% OSM transcript (blue curves) for kidney renal clear cell carcinoma (KIRC). Log-rank test p value, hazard ratio (HR), significance of hazard ratio p(HR) and number of patients are indicated on each plot. Panels were generated by using the GEPIA website in August 2025 (32).
